# International phase IV validation study of an EORTC quality of life questionnaire for testicular cancer patients: the EORTC QLQ-TC26

**DOI:** 10.1186/s12885-018-5036-8

**Published:** 2018-11-12

**Authors:** Monika Sztankay, Neil K. Aaronson, Juan I. Arraras, Umberto Basso, Uros Bumbasirevic, Fabio Efficace, Johannes M. Giesinger, Colin D. Johnson, Marieke van Leeuwen, Anne S. Oberguggenberger, Roman Sosnowski, Teresa Young, Bernhard Holzner, on behalf of the European Organisation for Research and Treatment of Cancer Quality of Life Group (EORTC QLG)

**Affiliations:** 1 Medical University of Innsbruck, University Hospital Innsbruck, Psychiatry II, Innsbruck, Austria; 2 Department of Psychiatry, Psychotherapy & Psychosomatics, Medical University of Innsbruck, Anichstrasse 35, Innsbruck, Austria; 3 Division of Psychosocial Research & Epidemiology, The Netherlands Cancer Institute, Amsterdam, The Netherlands; 4 Medical Oncology Department, Hospital of Navarre, Navarre, Spain; 5 Medical Oncology 1, Istituto Oncologico Veneto (IOV) IRCCS, Padova, Italy; 6 Clinic of Urology, Clinical Center of Serbia, University of Belgrade, Belgrade, Serbia; 7 Health Outcomes Research Unit, Italian Group for Adult Hematologic Diseases (GIMEMA), Rome, Italy; 8 University Surgical Unit, University of Southampton, Southampton University Hospitals, Southampton, UK; 9 Urooncology Department, Maria Sklodowska Curie Memorial Cancer Hospital, Warsaw, Poland; 10 Jackson Macmillan Centre, East & North Hertfordshire NHS Trust including Mount Vernon Cancer Center, Northwood Middlesex, UK

**Keywords:** Testicular cancer, Health-related quality of life, EORTC, Psychometric validation, Patient-reported outcome

## Abstract

**Background:**

Given the high cure-rate for testicular cancer (TC) and the patients’ young age, comprehensive evaluation of health-related quality of life (HRQOL) is an important consideration in this patient population. The EORTC QLQ-TC26 questionnaire module has been developed to supplement the EORTC QLQ-C30 in assessing TC-specific HRQOL in clinical trials and routine clinical practice. This international, multicentre phase IV validation study evaluated the psychometric properties of the new module.

**Methods:**

This international, multicentre phase IV validation study enrolled testicular cancer patients from seven European countries. Patients completed the EORTC quality of life core questionnaire EORTC QLQ-C30 and the QLQ-TC26 at two consecutive time points and a debriefing questionnaire regarding the QLQ-TC26 after baseline assessment. Psychometric evaluation included examination of the hypothesized module scale structure, internal consistency and test-retest reliability, known-groups validity, responsiveness to change over time and cross-cultural acceptability.

**Results:**

Data from 313 patients (mean age 38.6, SD 9.5) were analysed. All items exhibited a high completion rate with less than 2.4% missing values except for the sexuality items (up to 8.8%). The confirmatory factor analysis supported the hypothesised scale structure of the QLQ-TC26. Test-retest reliability was good for 8 of 12 scales (intraclass correlation: R t1|t2 ranged from 0.71–0.91) and four scales did not meet the acceptable criteria. Internal consistency was good for all twelve scales (Cronbach alpha = 0.79–0.90), except Communication (alpha = 0.67) and Sexual Functioning (alpha = 0.62). The module was able to distinguish clearly between patients with differing clinical status. Responsiveness to change over time was acceptable.

**Conclusion:**

The EORTC QLQ-TC26 is a valid, reliable and well-accepted condition-specific questionnaire, supplementing the EORTC QLQ-C30, for the assessment of testicular cancer patients’ HRQOL in clinical trials.

**Electronic supplementary material:**

The online version of this article (10.1186/s12885-018-5036-8) contains supplementary material, which is available to authorized users.

## Background

Testicular cancer (TC) is characterised by a low prevalence (less than 1 % of all male malignancies), a low mortality rate (less than 5 % within five years), a young patient population (about two thirds < 40 years) and increasing incidence rates [1, 2]. Mortality rates have dropped significantly over the past three decades due to more effective treatment options, shifting the focus of interest on the oncological treatment’s impact on patients’ health-related quality of life (HRQOL). Approximately 75–80% of patients with seminoma present with stage I disease, with a cancer-specific survival rate of 97–100% and a low recurrence rate (below 6%) under surveillance [3, 4]. Likewise, 60% of the patients with non-seminoma germ cell TC are diagnosed at an early stage having a 14–48% risk of recurrence within two years after surgery [5]. Treatment option encompass orchiectomy, retroperitoneal lymph node dissection with nerve sparing [6], chemotherapy with carboplatin (in early stage) [7] and Bleomycin, Etoposide and Cisplatin (for advanced disease) [8], as well as active surveillance [5].

Treatment strategies, although contributing to low mortality, are associated with various acute and long-term side effects [9]. These include gastrointestinal symptoms, peripheral neuropathy, Raynaud-like symptoms, hearing loss, tinnitus, heightened levels of fatigue, anxiety, cancer-related distress, sexual dysfunction, changes in body image and psychosocial problems [9–11]. Post-chemotherapy resection of residual masses can also result in chronic problems such as loss of antegrade ejaculation [12]. Survivors whose treatment included chemotherapy are at greater risk for long-term sequelae such as pulmonary toxicity, secondary malignant neoplasms, cardiovascular disease and decreased fertility [13]. Since the majority of TC survivors are at an age when fatherhood is a important consideration, fertility concerns and impaired sexual functioning may especially affect patients’ HRQOL, as well as that of their partners [14, 15].

Given the high cure-rate and the patients’ young age, evaluation of long-term HRQOL is an important consideration [16]. Previous studies comparing the HRQOL of men with TC with that of their general population peers have tended to use generic HRQOL questionnaires. These studies have typically reported similar levels of HRQOL for TC survivors and healthy men from the general population [17–20]. Generic HRQOL measures may, however, fail to assess symptoms and functional limitations specific to the TC patient population (e.g. infertility, body image and sexuality) and exhibit ceiling effects reducing the instruments’ sensitivity and discriminant validity [16, 19]. Standardising assessment of HRQOL with instruments developed according to rigorous questionnaire development guidelines guaranteeing a TC patient-centred approach, cross-cultural applicability and compatibility with existent cancer-specific core measures will enable comparability of results across studies and TC patient populations.

The pilot work of Fosså and colleagues [21] was a first important step towards the development of comprehensive HRQOL assessment of TC patients. However, their questionnaire has not been taken beyond the pilot stage of development and testing. Hoyt and colleagues have developed and tested a version of the Cancer Assessment for Young Adults (CAYA) questionnaire for young men with TC [22]. While the psychometric properties of this questionnaire appear to be good, it is questionable whether such a lengthy questionnaire can be widely adopted for use in clinical trials and daily clinical practice, where the economy of assessment burden is important. The developers of CAYA, therefore, propose the independent use of subscales which was supported by psychometric evaluation.

The European Organisation for Research and Treatment of Cancer (EORTC) Quality of Life Group has long used a standardized methodology to develop generic and condition-specific HRQOL questionnaires [23]. In the current paper, we report on the development and testing of the QLQ-TC26 for assessing the HRQOL of men with TC. This questionnaire has been developed according to the stepwise guidelines of the EORTC for questionnaire module development. This involves four phases: I) generation of relevant QOL issues, II) operationalization of the QOL issues into a set of items, III) pre-testing the questionnaire module; and IV) larger scale, international field testing of the psychometric properties of the module. Phases I to III of the development of the QLQ-TC26 were carried out between 2006 and 2011 and have been published previously [24]. This paper presents results of the final phase IV of developing this module.

## Methods

### Sample

Patients were recruited using the following inclusion and exclusion criteria: (1) diagnosis of testicular cancer, with or without relapse, (2) age 18 years or older, (3) sufficient reading ability to understand the questionnaire in their native language, (4) no obvious cognitive impairments, (5) provision of written informed consent, and (6) not participating in another HRQOL-related investigation that might interfere with the study.

To include patients during treatment as well as post-treatment, patients were recruited from two distinct groups. The *on-treatment group (A)* included patients undergoing surgery (with or without subsequent chemotherapy) who were assessed one to three days after surgery (T1on) and four weeks later (T2on). Although the protocol also allowed for recruitment of patients undergoing surgery then radiotherapy, no such patients presented during the enrolment period. The *post-treatment group (B)* was composed of patients who were at least one year post-treatment (T1post). They were assessed again after one week (T2post) in order to investigate test-retest reliability.

Patients were recruited from seven European countries (Austria, Italy, the Netherlands, Poland, Serbia, Spain, and the UK). Ethics committee approval was obtained at participating centres, following national requirements. Written informed consent was obtained from all participants, as required. The protocol was approved by the EORTC Quality of Life Group.

### Assessment instruments

This study required completion of a case report form assessing clinical and sociodemographic data plus the following questionnaires and interview:

#### EORTC QLQ-C30

The EORTC QLQ-C30 [25] has been internationally validated and is one of the most widely used cancer-specific HRQOL-instruments. It is composed of 30 items organised into five functioning scales (physical, role, cognitive, emotional and social), a global QOL scale, three symptom scales (fatigue, nausea/vomiting and pain) and six single symptom items.

*EORTC QLQ-TC26:* Based on the results of Phase III of the development process [24], this testicular cancer-specific module includes 26 items organised into 7 multi-item scales and 6 single items addressing: treatment side effects (8 items), treatment satisfaction (2 items), future perspective (2 items), work/education problems (single item), physical limitations (single item), infertility (single item), family problems (single item), sexual activity (2 items), sexual enjoyment (2 items), sexual problems (2 items), communication (2 items), body image problems (single item) and testicular implant satisfaction (single item). Based on conceptual considerations the Job/ Education Problems scale hypothesised in phase III was split into two single-item scales (Job Problems and Physical Limitations) in phase IV.

The EORTC QLQ-TC26 is currently available in English, Dutch, German, Italian, Spanish, Serbian and Polish. Translations were performed according to the EORTC translation guidelines [26]. For both, the QLQ-C30 and the QLQ-TC26, high impairment is indicated by low scores for the functioning scales and high scores for the symptom scales.

#### Debriefing questionnaire

At T1, patients completed a debriefing questionnaire that assessed time required to complete the QLQ-TC26 and the QLQ-C30, whether any help was needed, and whether any of the items were upsetting, confusing or difficult to answer. Additional free text comments were invited.

### Data collection procedure

Baseline assessment was done in the respective collaborating hospital upon receipt of informed consent, whereas follow-up assessment was performed either in the hospital or web-based at home Electronic data capture was available by means of the Computer-based Health Evaluation System (CHES [27]). CHES is a web-based software program that enables electronic data assessment in routine practice and clinical trials. It allows multicentre study monitoring providing electronic case report forms and web-based assessment of clinical and patient-reported data. CHES has already been implemented in other phase IV studies of the EORTC Quality of Life Group for the purpose of international field testing (https://ches.eortc.be/cms/module.html).

Those centres willing and able (depending on ethical approval and information technology infrastructure) to participate in electronic data capture were provided with access to the CHES data collection website. The website provided forms for entering clinical and sociodemographic data and allowed patients to complete the EORTC QLQ-C30 and the EORTC QLQ-TC26 via desktop computer or a tablet-PC. For the follow-up assessment, patients were provided with a username and password to complete the questionnaires online at home.

Prior to implementing web-based patient-reported data collection, a cognitive debriefing questionnaire and usability testing for CHES were conducted [28]. At institutions not participating in electronic assessment, data collection was performed using paper-and-pencil versions of the questionnaires.

### Statistical analysis

*Scale structure, internal consistency and test-retest reliability:*

We conducted confirmatory factor analysis (CFA) to examine the hypothesised scale structure of the QLQ-T26.

We calculated standardised factor loadings for each item with regard to the corresponding scale and considered loadings above 0.40 to be sufficient [29]. Residual variance of single-item factors was set based on the test-retest reliability of the scales in the cancer group post-treatment (see below). Model-data-fit was assessed with the Comparative Fit Index and the Tucker-Lewis Index, with both indices considered to indicate good fit if they exceeded a value of 0.95 [30]. We also calculated the Root Mean Square Error of Approximation (RMSEA) as a further parameter for model-data-fit, with a value below 0.05 indicating a good fit [31].

We calculated Cronbach’s alpha coefficients to assess the internal consistency reliability of each scale. Values above 0.70 were considered acceptable for purposes of group comparison [32].

Test-retest reliability was assessed in the post-treatment group only using the intraclass correlation coefficient (ICC), as well as calculating the percentage of absolute agreement for each scale. An ICC above 0.70 was regarded as adequate [32].

#### Validity and responsiveness

To assess convergent validity, we examined the correlation between single- and multi-item scales of the EORTC QLQ-TC26 and selected scales of the EORTC QLQ-C30. Correlations above 0.50 were considered to indicate convergent validity and correlations below 0.30 to indicate discriminant validity [33]. It was expected that those scales conceptually related would correlate substantially with one another. These scales were the QLQ-TC26 scales Treatment Side-Effects and Treatment Satisfaction vs. the QLQ-C30 functioning and symptom scales, Future Perspective (QLQ-TC26) vs Emotional Functioning and Social Functioning (QLQ-C30), Family Problems (QLQ-TC26) vs Social Functioning (QLQ-C30),

Physical Limitations (QLQ-TC26) vs Physical and Role Functioning (QLQ-C30), Job and Educational Problems (QLQ-TC26) vs Role Functioning and Financial Impact (both QLQ-C30) as well as the Communication and the Body Image scales (both QLQ-TC26) vs. Emotional Functioning (QLQ-C30). Additionally, we hypothesised that the QLQ-TC26 scales assessing sexuality (Sexual Activity, Functioning and Enjoyment) would show moderate correlations with the QLQ-C30 scales. Therefore, we assumed that correlations with those scales would qualify neither for convergent nor for discriminant validity.

Given the scoring as indicated above, for the Treatment Side Effect scale (QLQ-TC26), negative correlations were expected with the QLQ-C30 functioning and positive correlations with the QLQ-C30 symptom scales.

Known-group validity was tested with Student’s t-test for independent samples, comparing patients on- and off-treatment and patients with metastatic versus non-metastatic disease. This was based on the assumption that patients off-treatment as well as without metastatic disease would perform better.

Responsiveness to change was analysed with linear mixed models comparing patients at the start of treatment and four weeks later.

The CFA was conducted with the software package R [34] using the “Iavaan” package [35]. All other analyses were done with SPSS 21.0.

## Results

### Sample characteristics

From end of 2012 to 2015, a prospective sample of 313 TC patients was recruited in the UK (*N* = 130), Austria (*N* = 93), Poland (*N* = 25), Italy (*N* = 22), the Netherlands (*N* = 20), Serbia (*N* = 14) and Spain (N = 9). 113 patients were still receiving (on-treatment group), 200 have already completed treatment (post-treatment group).

Mean age in the on-treatment group was 36.1 years (SD 9.9), in the post-treatment group 40.1 years (SD 9.0). Mean time since diagnosis was 6.7 months (SD 17.7) for patients on treatment, 5.4 years (SD 3.4) for patients post-treatment. About half of the patients were married or cohabitating and had at least 14 years of education (52.7%). Most patients (76.4%) were working full-time at the time of the assessment and had never been unemployed during the treatment phase (72.1%). Forty-two percent did not have children, 20.3% had one child, and 37.7% had two or more children. Most patients had non-metastatic disease (81.4%), no recurrence (85.8%) and a diagnosis of seminoma (62.5%). At the time of the assessment, 63.9% of the patients were post-treatment (> 12 months) and 36.1% were receiving treatment (84.1% surgery and 85.2% chemotherapy). Further details are given in Tables 1 and 2. Missing sociodemographic and clinical data were acquired from the respective clinical information systems.

**Table 1:** Demographics of the patient sample (*n* = 313)

		Number	Percent
Age: mean (SD)	38.6 (9.5)		
Education			
	10 years or less	28	10.3
	11–13 years	101	37.0
	14 years or more	144	52.7
	Missing	40	
Marital Status			
	Never married	113	38.0
	Married or cohabiting	147	49.5
	Separated/divorced	19	6.4
	Other	18	6.1
	Missing	16	
Employment Status			
	Full-time	210	76.4
	Part-time	15	5.5
	Retired	8	2.9
	In professional training	4	1.5
	Sick leave	8	2.9
	Unemployed	17	6.2
	Other	13	4.7
	Missing	38	
Unemployment during treatment			
	Yes	78	27.9
	No	202	72.1
	Missing	33	
Number of children			
	0	116	42.0
	1	56	20.3
	2	74	26.8
	3	24	8.7
	4	6	2.2
	Missing	37	

Percentages are given for valid cases

**Table 2:** Clinical patient characteristics

		Number	Percent
Treatment status	On treatment	113	36.1
	Post treatment	200	63.9
Time since diagnosis [in months]	On treatment	mean 6.7	SD 17.7
	Post treatment	mean 5.4	SD 3.4
Histological tumour type	Seminoma	162	62.5
	Non-Seminoma	97	37.5
	Missing	54	
Metastases	Metastases Yes	50	18.6
	Metastases No	219	81.4
	Missing	44	
Recurrence	Recurrence Yes	39	14.2
	Recurrence No	235	85.8
	Missing	39	
Desire for a child	No	143	55.6
	Yes	114	44.4
	Missing	56	
Testicular Implant	No	182	75.2
	Yes	60	24.8
	Missing	71	
On-treatment Group: Surgery	Surgery No	14	15.9
	Surgery Yes	74	84.1
	Missing	25	
On-treatment Group: Chemotherapy	Chemotherapy No	13	14.8
	Chemotherapy Yes	75	85.2
	Missing	25	
Post-treatment Group: Previous Surgery	Surgery No	9	5.5
	Surgery Yes	154	94.5
	Missing	37	
Post-treatment Group: Previous Chemotherapy	Chemotherapy No	60	36.1
	Chemotherapy Yes	106	63.9
	Missing	34	
Post-treatment Group: Previous Radiotherapy	Radiotherapy No	157	94.0
	Radiotherapy Yes	10	6.0
	Missing	33	

Percentages are given for valid cases

### Compliance rates and debriefing results

The dataset was screened for missing responses to the scoring items. Across all time points the largest percentage of missing responses was observed for items assessing sexuality (item 25: 8.8% missing, item 24: 8.1%, item 23: 6.3%, item 22: 6.1%, and item 21: 1.1%). For items assessing treatment satisfaction percentage of missing responses was 2.4% (item 9) and 2.2% (item 10) respectively. All other items had less than 1% missing responses.

Of the 313 patients, 173 completed the debriefing questionnaire at the baseline assessment. Questionnaire completion required, on average, 8.1 min (SD 4.2; range two to 30 min). Only 6% of the patients required any help from family members or health care staff with completing the questionnaires. Twelve percent of the patients who completed the debriefing questionnaire indicated that at least one question was confusing or difficult to answer, and 3 patients found at least one upsetting. Comments applied primarily to questions related to sexual activity, which were perceived as sensitive but relevant.

### Usability testing of the CHES software for ePRO assessment

Overall, 48% of all assessments were conducted electronically. Assuming similarity in reception and usage of ePRO in this comparably young patient group, a consecutive subsample was questioned on the usability of electronic PRO assessment at the lead site in Austria (*n* = 15). Respondents (mean age 35.5 years, SD 7.5.; range 27–49) were highly educated (*n* = 12, 80% with more than 11 years of education), mainly post-treatment (n = 12, 80%) and without metastases or recurrent disease. All respondents successfully navigated through the online questionnaire (e.g., skip questions, changing responses if so desired, proceeding to the next question, saving responses, etc). Three respondents reported minor issues concerning the visual display of the electronic questionnaire features (e.g., suggested larger font size and different icon colouring).

### Factor structure and reliability of the QLQ-TC26

In the confirmatory factor analysis, all standardised factor loadings exceeded the threshold of 0.40 (0.621–0.977), confirming the hypothesised scale structure of the QLQ-TC26. The comparative fit index (CFI) = 0.974, the Tucker-Lewis fit index (TLI) = 0.963, and the RMSEA = 0.046 indicated good fit of the model and the data. Standardised factor loadings are provided in Table 3.

**Table 3:** Internal consistency, test-retest reliability and standardised factor loadings for the QLQ-TC26

Scale	Cronbach’s Alpha	Test-retest ICC	Item #	Standardised Factor Loading
Treatment Side-effects	0.80	0.91	Item 1	0.752
			Item 2	0.776
			Item 3	0.769
			Item 4	0.721
			Item 5	0.752
			Item 6	0.621
			Item 7	0.711
			Item 8	0.624
Treatment Satisfaction	0.87	0.48	Item 9	0.977
			Item 10	0.912
Future Perspective	0.79	0.71	Item 11	0.889
			Item 12	0.852
Job and Education Problems		0.81	Item 13	0.810
Physical Limitation		0.67	Item 14	0.670
Family Problems		0.65	Item 15	0.650
Infertility		0.82	Item 16	0.820
Communication	0.67	0.80	Item 17	0.708
			Item 21	0.944
Body Image Problems		0.75	Item 18	0.750
Sexual Activity	0.81	0.84	Item 19	0.905
			Item 20	0.825
Sexual Functioning	0.62	0.89	Item 22	0.780
			Item 23	0.765
Sexual Enjoyment	0.90	0.73	Item 24	0.955
			Item 25	0.954
Testicular Implant Satisfaction^a^		0.69	Item 26	

^a^This conditional scale has been excluded from the confirmatory factor analysis

RMSEA = 0.046; CFI = 0.974; TLI = 0.963

The threshold for test-retest reliability (0.70) was exceeded for all scales (0.71–0.91), except for Physical Limitations (0.67), Family Problems (0.65), and Treatment Satisfaction (ICC = 0.48). The conditional scale Testicular Implant Satisfaction (0.69) could only be analysed in a subgroup of patients (*n* = 93).

For the multi-item scales, Cronbach’s alpha coefficients ranged 0.62 for Sexual Functioning to 0.90 for Sexual Enjoyment. All but two scales (Communication and Sexual Functioning) exceeded the 0.70 criterion for group level use (see Table 3 for further details).

Each item showed the strongest correlation with the designated scale.

### Convergent and discriminant validity

The Treatment Side-Effects scale showed strong correlations (all *p* < 0.001) with the QLQ-C30 scales for Physical Functioning (*r* = − 0.55), Role Functioning (*r* = 0.50), Social Functioning (*r* = − 0.50), Emotional Functioning (*r* = − 0.51), Global QOL (r = − 0.55), Fatigue (*r* = 0.65) and Pain (*r* = 0.51), while the Treatment Satisfaction scale did not correlate significantly with any of the QLQ-C30 scales (all *r* < 0.07).

For Future Perspective, the hypothesised correlation with Emotional Functioning failed to exceed the threshold for convergent validity of 0.50 (*r* = 0.49, *p* < 0.001). In addition, the single item concerning Family Problems did not show the expected correlation with Social Functioning (*r* = − 0.47, *p* < 0.001) and the Body Image scale failed to exceed the threshold for convergent validity with Emotional Functioning (*r* = − 0.44, *p* < 0.001).

Job and Education Problems showed high correlations with Role Functioning (*r* = − 0.52) and Financial Impact (*r* = 0.62, p < 0.001). Physical limitations showed convergent validity (all p < 0.001) with Physical Functioning (r = − 0.55), Role Functioning (*r* = − 0.67) and Global QOL (*r* = − 0.53).

Correlation between Communication and Emotional Functioning just exceeded the 0.30 cut-off for discriminant validity (*r* = 0.32, p < 0.001). All other correlations with the QLQ-C30 scales were low (*r* < 0.27). The single item on Infertility did not correlate significantly with any of the QLQ-C30 scales (all *r* < 0.23).

### Known groups validity and responsiveness

Known group comparisons were performed between patients on- and off-treatment and patients with metastatic versus non-metastatic disease. Comparing patients four weeks after start of treatment (*T2on*) with those in the post-treatment phase (*T2post*), we found the largest differences for Sexual Activity (40.5 vs 63.7, p < 0.001), Physical Limitations (36.1 vs 15.3, p < 0.001), Family Problems (34.7 vs 22.8 points, *p* = 0.012), Body Image Problems (23.6 vs 12.8 points, *p* = 0.039) and Treatment Side-Effects (17.1 vs 9.3, *p* = 0.002). All differences were in favour of patients in the post-treatment phase.

The largest mean differences between patients post-treatment with and without previous metastatic disease were found for Sexual Enjoyment (59.3 vs 81.3 points, *p* = 0.001), Sexual Activity (50.0 vs 67.9 points, *p* = 0.004) and Communication (71.4 vs 85.0 points, *p* = 0.013). The difference in Treatment Satisfaction failed to reach statistical significance (78.7 vs 92.7 points, *p* = 0.079). Further details are given in Table 4.

**Table 4:** QLQ-TC26 scores in patients with previous metastatic or non-metastatic disease post-treatment

	Previous metastatic disease *N* = 21		Previous non-metastatic disease *N* = 157			
EORTC QLQ-TC26	Mean	SD	Mean	SD	t-statistic	*p*-value
Treatment Side Effects	15.3	21.8	8.2	12.3	1.461	0.158
Treatment Satisfaction	78.7	31.2	92.7	19.7	−1.860	0.079
Future Perspective	65.9	26.1	67.3	26.6	−0.233	0.816
Family Problems	30.0	34.0	21.4	29.0	1.218	0.225
Infertility	30.2	31.5	29.9	33.8	0.029	0.977
Communication	71.4	26.9	85.0	22.8	−2.510	0.013*
Body Image Problems	22.2	32.2	11.3	20.5	1.520	0.143
Sexual Activity	50.0	26.4	67.9	26.3	−2.932	0.004**
Sexual Functioning	76.9	33.9	87.7	21.8	−1.325	0.201
Job Education problems	22.2	35.5	10.0	23.7	1.536	0.139
Physical limitations	22.2	35.5	13.0	24.4	1.161	0.258
Sexual Enjoyment	59.3	32.5	81.3	24.3	−3.503	0.001**
Testicular Implant Satisfaction	66.7	38.5	66.7	32.9	< 0.001	1.000

Note. *p <.05, **p <.01

In the on-treatment group, the largest changes between the assessment at the start of treatment (*T1on*) and after four weeks (*T2on*) were found for Job and Education Problems (improvement from 32.4 to 16.1 points, p = 0.001), Sexual Activity (deterioration from 54.9 to 40.5 points, p = 0.002), Body Image (deterioration from 13.6 to 23.6 points, *p* = 0.016), Sexual Enjoyment (deterioration from 78.6 to 70.6 points, *p* = 0.025) and Treatment Side-Effects (deterioration from 12.3 to 17.1 points, p = 0.002). Please see Table 5 for further details.

**Table 5:** QLQ-TC26 scores at the start of treatment, the end of treatment and post-treatment

	On-Treatment							Post-Treatment			
	T1on *N* = 113		T2on *N* = 49			T1on vs T2on^a^		T1post *N* = 200		T2on vs T1post^a^	
EORTC QLQ-TC26	Mean	SD	Mean	SD	Diff.	F-statistic	p-value	Mean	SD	t-statistic	p-value
Treatment Side Effects	12.3	15.1	17.1	15.2	4.8	10.841	0.002**	9.3	13.9	3.252	0.002**
Treatment Satisfaction	89.1	21.0	91.2	16.4	2.1	0.687	0.410	90.4	22.7	0.230	0.818
Future Perspective	61.9	27.4	65.3	23.0	3.4	0.339	0.563	66.9	26.9	−0.386	0.700
Family Problems	36.6	34.2	34.7	27.5	−1.9	0.481	0.491	22.8	29.7	2.537	0.012*
Infertility	36.6	37.3	27.9	31.4	−8.7	2.824	0.099	28.5	33.5	−0.115	0.908
Communication	81.7	26.1	83.7	23.9	2.0	1.022	0.316	82.3	24.1	0.350	0.727
Body Image Problems	13.6	23.8	23.6	33.7	10.0	6.124	0.016*	12.8	22.3	2.110	0.039*
Sexual Activity	54.9	29.0	40.5	31.4	−14.4	10.636	0.002**	63.7	27.2	−5.178	< 0.001**
Sexual Functioning	91.3	20.2	91.7	16.0	0.4	0.027	0.871	86.4	23.2	1.646	0.105
Job Education problems	32.4	35.8	16.3	24.6	−16.1	7.043	0.011*	12.5	26.6	0.915	0.361
Physical limitations	32.4	33.5	36.1	29.5	3.7	1.054	0.308	15.3	27.1	4.710	< 0.001**
Sexual Enjoyment	78.6	27.0	70.6	28.4	−8.0	5.410	0.025*	78.4	25.6	−1.613	0.108
Testicular Implant Satisfaction	45.8	40.4	56.9	42.1	11.1	0.391	0.541	66.7	34.3	−0.973	0.334

^a^T1on - first assessment time point in the on-treatment group before treatment start; T2on - second assessment time point in the on-treatment group at the end of treatment; T1post - first assessment time point in post-treatment group;

*p< .05, **p< .01

## Discussion

In this international phase IV field study, we evaluated the psychometric characteristics of the EORTC QLQ-TC26 module in a sample of 313 TC patients. In line with the EORTC Module Development Guidelines, these patients were from Southern, Western, Northern and Eastern Europe. No item was removed or changed from the phase III module. Based on conceptual considerations the Job/ Education Problems scale hypothesised in phase III was split into two single-item scales (Job Problems and Physical Limitations) in phase IV. The final QLQ-TC26 (see Additional file 1) comprises seven multi-item scales (treatment side effects, treatment satisfaction, future perspective, communication, sexual activity, functioning and enjoyment) and six single items (job and education problems, physical limitations, family problems, infertility, body image problems, testicular transplant satisfaction).

The hypothesised scale structure of the QLQ-TC26 was supported by confirmatory factor analysis and satisfactory internal consistency. All but two scales (Communication and Sexual Functioning) exceeded the 0.70 criterion for group level use concerning internal consistency. The two items of the Communication scale were found to be interdependent, indicating that patients able to talk about the disease were mostly able to talk about sexuality, but not necessarily the other way round. For Sexual Functioning a substantial proportion of patients reported no impairments at all, while those reporting issues frequently had either problems with ejaculation or with erection, but not always both. Patients did comment on the sensitive nature of items about sexuality, but agreed that these items were relevant. Percentage of missing items on sexuality is comparable or even lower than in other EORTC QOL modules [36].

The test-retest reliability analysis of the questionnaire met the ICC threshold for excellent reliability (0.90) for the Treatment Side-Effects scale. The threshold for good reliability of 0.70 was almost met or exceeded for the remaining multi-item scales, except the Treatment Satisfaction scale which exhibited the lowest test-retest reliability. This might be related to the issue of discontinuation of clinical care and a change to self-managed follow-up.

While internal consistency measured by Cronbach Alpha coefficients was not good for all scales, the structural equation model showed good factor loadings of the individual items on the respective scales supporting the overall scale structure of the module.

Most of the hypotheses on correlations between the QLQ-TC26 and the QLQ-C30 scales could be confirmed which indicates good convergent and discriminant validity for the QLQ-TC26 scales. Correlations for the Family Problems (vs. Social Functioning) and Body Image scale (vs. Emotional Functioning) closely missed the predefined thresholds for convergent validity. For Family Problems, this may relate to the fact that the QLQ-C30 scale measures actual impairment of social relations, whereas the QLQ-TC26 assesses patients’ concerns about possible problems in this area. For the Communication scale correlations with the QLQ-C30 scales indicated discriminant validity as expected, with the exception only of the correlation with Emotional Functioning that slightly exceeded the threshold. Furthermore, the QLQ-C30 does not cover issues concerning sexuality which are, however, relevant for TC patients.

Known-group analyses confirmed the ability of the QLQ-TC26 to discriminate between subgroups of patients who differed clinically with regard to treatment status and extent of disease. As expected, patients in long-term follow up had better scores in the scales Treatment Side-Effects, Family Problems, Body Image Problems, Sexual Activity and Physical Limitations when compared to patients at the end of treatment. This is in accordance with studies showing that HRQOL of TC survivors improves after treatment [19]. In our study, metastatic disease, however, results in higher impairment in Sexual Enjoyment, Sexual Activity as well as Communication in patients with metastatic disease post-treatment.

Changes of scores were hypothesised in the course of the treatment trajectory and were broadly congruent with clinical expectations. When comparing patients’ HRQOL before start of treatment and four weeks later, results indicated deterioration in Body Image, Sexual Activity, Sexual Enjoyment and Treatment Side Effects, while Job and Educational Problems improved following diagnosis. It might be hypothesised that the latter may be the result of increased adaptation to the situation, after having dealt with administrative aspects such as sick leave and the engagement of supportive services.

Based on the results of this phase IV study, assessment with the QLQ-TC26 is recommended when investigating changes in disease-specific HRQOL across the treatment trajectory in clinical trials or clinical routine. It is also appropriate for assessing the HRQOL of patients in the early post-treatment survivorship period (defined herein as up to 1 year following completion of treatment). Where the focus of a study is on the HRQOL of longer term TC survivors, another type of survivorship questionnaire may be needed. The EORTC Quality of Life Group is currently developing a questionnaire for assessing the QOL of cancer survivors including those suffering from TC. In the previous phases of the questionnaire development, it was decided not to define treatment phase-specific scales as this would have complicated and limited the consistency of longitudinal QOL assessment from diagnosis to follow-up [24]. Assessments of the HRQOL in young adults might benefit from combinations with available questionnaires such as the CAYA [22].

A limitation of this study was that patients treated with radiotherapy were only included in the off-treatment group, which might be attributable to the current clinical recommendations, proposed by international guidelines [7]. Published data on long-term toxicity and the increased risk for second non-germ cell malignancies indicate that adjuvant radiotherapy should no longer be recommended as first-line adjuvant treatment for patients with stage I seminoma [37] and younger patients [7]. It is, however, still considered as a treatment option in patients with TC stage IB, IIA and IIB, though, diagnosis at the latter stage is less common. Such is metastatic disease in TC patients, resulting in a limited subsample.

Missing data might be explained by the fact that extraction of missings from the clinical information systems was of varying success across participating centres. Another issue was the set-up of the clinical report form. Several variables had to be derived from the patients themselves (e.g. marital status) and could not be acquired from the medical charts. To reduce the odds for missings, these parts of the clinical report form (meant to be completed by the study assistant approaching the patient) should have been administered as part of the patient questionnaire.

Despite the above mentioned limitations, with regard to the scale structure, the current structure is well-defined from a conceptual point and it was supported empirically by the structural equation model. While the EORTC QLQ-TC26 has begun to see use in clinical trials [EudraCT2014–003930-17] [NCT02304575], future studies should aim for further application of the EORTC QLQ-TC26 to assess utility of the measure in more versatile cultural settings (e.g. in non-European regions and in ethnic minorities) and to evaluate its applicability in a more dynamic assessment approach supplemented with relevant validated subscales.

The strengths of this study are the systematic development and validation across various linguistic and cultural contexts, reflecting the rigorous EORTC module development guidelines, The QLQ-TC26 assesses not only somatic, but also psychological and psychosocial issues in order to provide a comprehensive overview of TC patients’ disease- and treatment-related HRQOL. Patient feedback from the debriefing questionnaire indicated that the large majority of patients did not have any difficulties with and were not confused by the items, nor did they find the questionnaire items upsetting. Finally, the availability of an electronic version of the questionnaire using the CHES software can reduce the resources required for data collection and entry, and minimise data entry errors. Usability testing in a subsample of participating patients indicated that patients have few if any problems in completing the questionnaire digitally.

## Conclusion

The results of this international phase IV study provide evidence for the reliability and the validity of the EORTC QLQ-TC26. We recommend it to be used in conjunction with the QLQ-C30 in clinical trials as well as in daily clinical routine for the assessment of HRQOL across the treatment trajectory.

## Additional file

Additional file 1: Specimen EORTC QLQ-TC26 (PDF 28 kb)

## Abbreviations

CFA: Confirmatory factor analysis
CFI: Comparative fit index
CHES: Computer-based Health Evaluation System
EORTC QLQ-C30: European Organisation of Research and Treatment of Cancer Quality of Life Questionnaire Core 30
EORTC QLQ-TC26: European Organisation of Research and Treatment of Cancer Quality of Life Questionnaire Testicular Cancer 26
ePRO: Electronic patient-reported outcome
HRQOL: Health-related quality of life
ICC: Intraclass correlation coefficient
RMSEA: Root Mean Square Error of Approximation
