# Supplementary material for: International phase IV validation study of an EORTC quality of life questionnaire for testicular cancer patients: the EORTC QLQ-TC26
Source: BMC Cancer. 2018 Nov 12;18:1104. doi: 10.1186/s12885-018-5036-8 (PMC6233273; doi:10.1186/s12885-018-5036-8)
Supplement: Supplementary file 1 — Specimen EORTC QLQ-TC26 (PDF 28 kb) [file 12885_2018_5036_MOESM1_ESM.pdf]

EORTC QLQ-TC26

Patients sometimes report the following symptoms or problems. Please indicate the extent to which you have experienced these symptoms or problems during the past week. Please answer by circling the number that best applies to you.

During the past week :	Not at All	A Little	Quite a Bit	Very Much
31. Have you lost any hair?	1	2	3	4
32. Have you had problems with your sense of taste or smell?	1	2	3	4
33. Have you had pain in your stomach area?	1	2	3	4
34. Have you had acid reflux?	1	2	3	4
35. Have you had tingling or numbness in your fingers or toes?	1	2	3	4
36. Have you had skin problems (e.g. itchy, dry)?	1	2	3	4
37. Have you had pale/cold fingers or toes?	1	2	3	4
38. Did you have problems with hearing?	1	2	3	4
39. Were you satisfied with the medical care you received?	1	2	3	4
40. Were you satisfied with the information you received about your disease or treatment?	1	2	3	4
41. Did you feel uncertain about the future?	1	2	3	4
42. Have you been anxious about a possible recurrence of the disease?	1	2	3	4
43. Have you had any problems with your job or your education because of your disease or treatment?	1	2	3	4
44. Have you been physically limited as a result of your disease or treatment?	1	2	3	4
45. Were you concerned about disruption of family life?	1	2	3	4
46. Were you concerned about your ability to have children?	1	2	3	4
47. Can you talk about your disease with your partner or the person who is closest to you?	1	2	3	4

Please go on to the next page

SPECIMEN

**During the past week :**	**Not at All**	**A Little**	**Quite a Bit**	**Very Much**
48. Have you felt less masculine as a result of your disease or treatment?	1	2	3	4
49. To what extent were you interested in sex?	1	2	3	4
50. To what extent were you sexually active? (with or without intercourse)	1	2	3	4
51. Can you talk about sexuality with your partner or the person who is closest to you?	1	2	3	4
**Next questions only in the case of sexual activity:**				
52. Did you have difficulty getting or maintaining an erection?	1	2	3	4
53. Did you have problems with ejaculation?	1	2	3	4
54. To what extent was sex enjoyable for you?	1	2	3	4
55. Has the sexual relationship with your partner been satisfying?	1	2	3	4
**Answer this question only if you have a testicular implant:**				
56. Are you satisfied with your testicular implant?	1	2	3	4

SPECIMEN

© Copyright 1994 EORTC Quality of Life Group. All rights reserved. Version 1.0
